# Treatment of Delirium Tremens by Digitalis

**Published:** 1861-09

**Authors:** G. M. Jones


### SELECTIONS.

*Treatment of Delirium Tremens by Digitalis.* By G. M. JONES, Esq., Dr. WILLIAMS, and Dr. WEBSTER.

[Mr. Jones, of Jersey, treats these cases on a novel plan. His opinion is always valuable. From most other people we should dissent if they gave such an opinion, but Mr. Jones is truly trustworthy. He was led to give Digitalis from mere accident. He says :]

As to the dose, experience has taught me that the best dose is *half an ounce* of the tincture given in a little water. In some few cases this one dose is enough, but generally a second dose is required four hours after the first. In some cases, but very seldom, a third dose is called for ; but this hardly need ever exceed two drachms. The largest quantity I have ever given was *half an ounce* at first, *half an ounce* four hours afterwards, and another *half an ounce* six hours after that—making an ounce and a half in ten hours.

As to the effect of these doses, my impression is that the action is on the brain, not on the heart. The pulse, so far from being lowered in force, becomes fuller, and stronger, and more regular, soon after the first dose. The cold clammy perspirations pass off, and the skin becomes warmer. As soon as the remedy produces its full effect, sleep for five, six, or seven hours, commonly follows; sleep is the guide as to the repetition of the dose. No action on the kidneys is evidenced by any unusual secretion of urine. Sometimes the bowels are slightly acted on, but not commonly. I have never once seen any alarming sympton follow the use of these large doses of digitalis. The only case I have lost since adopting this treatment had a tumour in the brain.—In three only was other treatment adopted after digitalis had failed to procure sleep; in other words, in sixty-seven out of seventy cases, digitalis was the only medicine used, and sixty-six of these patients recovered. I do not mean that these are the exact numbers of those treated; I am certain as to the death, but I may have had more recoveries. I am well within bounds in saying seventy cases in twelve years, and that all of them were well-marked cases of delirum tremens. Slight cases of nervous derangement after drinking I have seen in great numbers; but I speak here only of such cases as required active treatment. My previous experience of the results of the treatment by opium, or some of its preparations, by anti-spasmodics, &c., had certainly been much less successful; the proportion of deaths was larger, and the recovery much less rapid. Again; I have treated more than one patient successfully by digitalis, who, in subsequent attacks elsewhere, has been treated by opium, and died; and in many of the cases in which I have used digitalis successfully, opium had been previously given without any good effect.

I will only allude to one case in illustration:—On September 9th, 1860, I was called to see a gentleman, 48 years of age, who was in a very alarming state, having been without sleep four days and nights, having been "muddled" for two months before, and having previously had "fits of the horrors." He had been treated by another practitioner by opium in moderate doses, but had become worse, and when I was sent for, it was the opinion of Mr. Spencer Wells and Mr. McCrea—who accompanied me in my first visit—that the case was as bad a one as *they* had ever seen; certainly, *I* never saw a worse. The pulse was almost imperceptible; the skin covered with cold, clammy perspiration; the face deadly pale; the lips blue; the hands tremulously grasping the air; the eye expressive of great fear; the mind gone; he was muttering incoherently. With some difficulty I passed half an ounce of tincture of digitalis down his throat in the presence of my friends. In a few minutes he became more tranquil, the pulse was felt more easily, and we left him.—After four hours I found that he had not slept, but he was rather more sensible, less tremulous, and warmer. I accordingly repeated the dose. Three hours after that, as he had been without sleep, though in other respects improving, I gave two drachms more, making ten drachms in seven hours. After this he had some sleep, and had slept at intervals during the night. The next morning Dr. Ballard saw him, with my other friends, and all of them were much pleased with the great improvement manifested. He was sensible, his fears had disappeared, he was very slightly tremulous; the skin was warm, the tongue moist, and the pulse full and regular at 90. The heart's sound and impulse were normal; the bowels had acted once, and urine had been passed in natural quantity. After this he took some broth, drank freely of imperial and lemonade, but took no stimuli of any kind, or any other medicine. He slept uninterruptedly for three hours and a half in the afternoon, and at intervals in addition. The next night was a good one; and when he was seen by my friends again the next morning he was almost well, and calling out for a mutton-chop.

I trust that this narrative of the results of my experience may induce others to follow what I believe to be a very valuable practical lesson; but I must warn those who do so not to try, as I have done, any smaller doses than those I have recommended. They would not only lose valuable time by so doing, but I believe would do harm. Doses of half a drachm or a drachm do no good whatever; and the pulse, in some cases where I tried them, became intermitting. I have never seen this effect from the larger doses; on the contrary, a feeble intermitting pulse has generally soon become fuller and more regular, proving, I think, as I said before, and as I again wish to impress on the profession, that the curative action is on the nervous system primarily, and not on the organs of circulation.—*Med. Times and Gazette, Sept.* 29, 1860, *p.* 301.

[Dr. Ballard, of Islington, corroborates Mr. Jones' opinion, and states as follows what he saw in two other cases, one in the hospital, and another in private lodgings.]

The former was a man apparently about fifty years of age, the other a young man about twenty-five or twenty-six years. Both had been drinking hard up to the period of seizure. In both, the characteristic symptons of the disease were clearly marked, the skin cold, and the pulse very feeble; in the hospital patient scarcely perceptible. In both cases, the earliest operation of the medicine was seen in the abatement of the nervous phenomena, in greater quietness of manner, and in the disappearance of delusion. Coincident with this the warmth returned to the surface, the pulse resumed its natural fulness, and a healthy perspiration appeared upon the skin. After this improvement had proceeded to a certain extent, both patients slept, but I could not regard the sleep as anything but an index of the favorable progress of the case. The improvement in the general condition clearly did not depend upon its production; first, because marked amendment had preceded it, and, next, because it was not prolonged beyond a very few hours. In no one of the three cases was there any unnatural increase in the quantity of urine passed. My surprise at witnessing the success of this remarkable treatment was only equalled by my admiration of the boldness and courage of the man who could introduce so startling an innovation of our practice. ' It certainly is not what any one would have expected: that feebly-acting heart would be roused into normal action by a medicine like digitalis; yet this is one of its earliest effects in the dose given by Mr. Jones.

From time to time very daring doses of digitalis have been administered. Dr. Pereira, in his "Elements of Materia Medica," mentioned the practice of Mr. King, of Saxmundham, who was in the habit of giving, in accute inflammation, doses of the tincture amounting to half an ounce or an ounce, without the production of any dangerous symptons. A pupil of Mr King once informed me, however, that during this treatment the maintenance of the recumbent posture was rigidly enforced. In neither of the cases which I saw treated by Mr. Jones did any pains appear taken to secure this precaution. In truth, the action of the drug upon the circulation, when it is used in inflammatory disease and in delirium tremens, seems utterly different. The effect in the former case, according to Mr. King, is to subdue the pulse and to render it irregular, an operation confirmed by Dr. Pereira's own experience of large doses; in the latter the pulse increases in force and fulness.

My own impression, from what I have seen and heard from Mr. Jones, is that in tincture of digitalis we have a true *counter-poison*,—an addition to that class of mutually-antidotal poisons of which opium and belladonna have been the most recent examples. Alcohol is the remedy by which we counteract the depression produced by digitalis: the converse is also true,—digitalis is most remarkably an antidote to alcoholism.

At a meeting of the Medical Society of London, May 12, 1828, Dr. Thos. Williams related an instance of a drunken butcher having taken two ounces of the tincture of digitalis in two doses of an ounce each, in quick succession, without the slightest inconvenience ensuing. This is quoted by Dr. Pereira.

When first I heard of Mr. Jones's treatment, I naturally suspected the quality of his tincture; but on mentioning this, he assured me that he had employed tincture from a variety of sources, and among the rest from Apothecaries' Hall, and always with the same result.—*Med. Times and Gazette, Oct.* 13, 1860, *p.* 363.

[This practice of Mr. Jones is recommended also by Dr. A. Wynn Williams, whose opinion is as follows:]

That such doses are as a rule not dangerous when there is any unnatural excitement of the nervous centres, is fully borne out by the fact that even ounce doses of tincture of digitalis have been prescribed twenty years ago to ward off epileptic attacks, when there was any preliminary warning of their approach. I was under the impression that it was Dr. Billing who so ordered it, but I cannot now find that he did so in any of the editions of his works. In such cases I have been in the habit of prescribing it in half ounce doses for many years, and have found it by far the most efficacious remedy.

The following is a foot note, written by myself, many years ago, under the head of Epilepsy:—"I have certainly averted the impending paroxysm by administering very large doses of digitalis, which does not appear to be so unmanageable and uncertain in its effects in large doses as in continued small ones. I have given as much as half an ounce of the tincture on the threatened approach of a paroxysm, and never saw any ill effects arising from administering so large a dose. One patient always kept a draught of the above dose in the house."

There is one form of excitement of the brain which I have never had an opportunity of administering it in, namely, puerperal convulsions. In this formidable disease, I decidedly think it deserves a trial.—*Med. Times and Gazette*, *Oct.* 6, 1860, *p.* 342.

[Dr. WEBSTER, physician to the Northampton Infirmary, although he would, he says, be timid in adopting Mr. Jones's treatment by digitalis, gives the opinion and practice of Mr. Wood, of Heene, Worthing, who treated a case of this kind with large doses of digitalis. Dr. Webster says:]

Corroborative testimony to the innocuousness of such a dose of our usual pharmacopœial preparation of that tincture, in our climate, to the average constitutions of our patients, can from one or the other no doubt be supplied, (Dr. Wynn Williams has given us one;) and I would try to report a somewhat analogous case, mentioned to me by Mr. F. B. Wood, of Heene, Worthing, and until recently practising in this town, hoping that others may thereby be induced to furnish their quota of experience *pro* or *con*. Mr. Wood writes to me as follows:—

"In reference to a plan recently suggested in the *Medical*

*Times and Gazette*, by Mr. Jones of Jersey, of treating of delirium tremens by tincture of digitalis, in compliance with your wish I send you a narrative of the case, in which (many years ago) I treated a patient laboring under furious delirium, with, as I then thought, large doses of the same drug. I should commence by stating that I had never previously attended the patient, but had learnt from reliable authority, viz., his previous medical attendant, that the patient, in the six or seven preceding years had three or four similar violent attacks. The treatment then adopted had been—venesection pleno rivo from both arms at once, and frequently repeated; hundreds of leeches to the forehead, by twenties and twenty-fours at a time; large quantities of calomel, and long continued drastic purgation. There were also several, and severe relapses on each of these occasions; and in one instance, after an interval of three weeks from the first seizure. On the last two occasions, when convalescent, the patient was in such a low and melancholy state, that daily and nightly watching, for three or four weeks, had to be maintained, for fear of the commission of suicide. I was summoned to attend at the last attack, in October, 1844, or 1845, and the residence being several miles away from Northampton—and having been forewarned of the probable nature of the case —I went armed with two ounces of the tincture of digitalis. Upon my arrival I at once gave a tea-spoonful of the tincture, and repeated every hour for four doses (the second dose having been given in rather less time.) The patient then becoming much quieter, and circulation steadier, I reduced the dose to half a drachm every two, three, and at last four hours, during the remainder of the first twenty-four hours from the seizure. The patient had only two or three slight relapses, and these within the first four days. On the third day, when there was a slight relapse, the physician who had previously attended was summoned in consultation. He applied twelve leeches to the temples and forehead, continued the digitalis, and ordered a fair quantity of calomel and colocynth. This was all the blood withdrawn on this occasion, and I confess even that against my judgment. However, when convalescence was established, there were none of the old symptoms of debility, melancholy, &c., as in previous attacks. Altogether the patient took about fourteen drachms of the tincture of digitalis, in the first four hundred hours of his illness."

As this patient has been under my supervision from time to time since Mr. Wood left this town, I can corroborate the fact of there having been no return of the complaint, even though there has too often been a sufficiently adequate amount of the accustomed provocation.—*Med. Times and Gazette, Oct.* 13, 1860, *p.* 364.—*Braithwaites Retrospect.*

